# Supplementary material for: Thresholds of lake and reservoir connectivity in river networks control nitrogen removal
Source: Nat Commun. 2018 Jul 17;9:2779. doi: 10.1038/s41467-018-05156-x (PMC6050239; doi:10.1038/s41467-018-05156-x)
Supplement: Supplementary file 1 — Supplementary Information [file 41467_2018_5156_MOESM1_ESM.pdf]

SUPPLEMENTARY INFORMATION

Thresholds of lake and reservoir connectivity in river networks control nitrogen removal

Noah M. Schmadel[1*], Judson W. Harvey[1], Richard B. Alexander[1], Gregory E. Schwarz[1], Richard B. Moore[2], Ken Eng[1], Jesus D. Gomez-Velez[3], Elizabeth W. Boyer[4], and Durelle Scott[5]

[1]U.S. Geological Survey, Reston, Virginia, USA, [2]U.S. Geological Survey, Pembroke, New Hampshire, USA, [3]Department of Civil and Environmental Engineering, Vanderbilt University, Nashville, Tennessee, USA, [4]Department of Ecosystem Science and Management, Pennsylvania State University, Pennsylvania, USA, [5]Department of Biological Systems Engineering, Virginia Polytechnic Institute and State University, Blacksburg, Virginia, USA

*Corresponding author: nschmadel@usgs.gov

Supplementary tables

Supplementary Table 1. Network characteristics and local and regional ponded water versus stream total nitrogen removal.

	Chesapeake Bay	New England	Total
**Network characteristics**			
Total number reaches (catchments)	84,214	107,042	191,256
Number of ponded water segments	6,225	22,821	29,046
Number of unique ponded waters	4,305	13,875	18,180
Total length of river network (km)	128,243	160,971	289,214
Network length covered by ponded waters (km)	2,956	15,427	18,383
Total drainage area (km^2)	169,650	270,878	440,528
Total area of ponded waters (km^2)	642	7,862	8,504
% of network length covered by ponded waters	2.3	9.6	6.4
Pond density (% area covered by ponded waters)	0.4	2.9	1.9
**Local ponded water versus stream removal**			
% pond-dominant (positive difference in proportion removed)	88.0	77.0	79.6
% stream-dominant (negative difference in proportion removed)	12.0	23.0	20.4
**Regional ponded water versus stream removal**			
% nitrogen removed, existing network (with ponded waters)	31.4	18.6	24.5
% nitrogen removed, stream only network (ponded waters replaced)	31.0	16.1	23.0
% of removed nitrogen load attributed to ponded waters	5.1	22.8	12.3
% of removed nitrogen load attributed to streams	94.9	77.2	87.7

Supplementary Table 2. Thresholds in pond density where ponded waters become dominant features to cumulative nitrogen removal.

Sub-region	Stream order	Pond density threshold (%)
Chesapeake Bay	1	0.46
	2	0.51
	3	0.71
	4	1.36
	5	0.64
	6	1.20
	7*	-
	Mean	0.81
New England	1	1.34
	2	1.95
	3	2.51
	4	2.56
	5	3.02
	6	2.15
	7	3.98
	Mean	2.50

*No threshold identified

Supplementary Table 3. Thresholds in pond connectivity where the dominance of nitrogen removal by ponded waters starts to decrease.

Sub-region	Threshold estimate	Standard error	95% confidence interval
Chesapeake Bay	0.186	0.034	0.119 to 0.254
New England	0.363	0.048	0.269 to 0.456

Supplementary Table 4. Percent variance explained by physical metrics important to assessing pond-stream dominance of nitrogen removal.

Sub-region	Linear regression	Variable	Degrees of freedom	Sum of squares	Mean square error	F	p	Percent variance explained
Chesapeake Bay	$R^2 = 0.67$	ln(pond connectivity)	1	416.5	416.5	1385.9	< 0.0001	12.4
	$p < 0.0001$	ln(circularity)	1	222.7	222.7	740.9	< 0.0001	6.6
	$F = 2490$	ln(drainage area)	1	1606.1	1606.1	5343.6	< 0.0001	47.8
		Residuals	3705	1113.6	0.3			33.2
New England	$R^2 = 0.55$	ln(pond connectivity)	1	1738.5	1738.5	2998.9	< 0.0001	11.8
	$p < 0.0001$	ln(circularity)	1	629.2	629.2	1085.3	< 0.0001	4.3
	$F = 4680$	ln(drainage area)	1	5770.9	5770.9	9954.6	< 0.0001	39.2
		Residuals	11378	6596.1	0.6			44.8

Supplementary Table 5. Summary of SPARROW model calibration results.

Total nitrogen, 2002 (n = 363, MSE = 0.1201, RMSE = 0.3465, R^2 = 0.969, yield R^2 = 0.829)

Explanatory variables	Coefficient units	Mean coefficient	Standard error	*p*-value*
**Sources**				
Point sources (kg y^{-1})	Dimensionless	1.179	0.205	< 0.0001
Crop fertilizer and fixation, corn soybeans, alfalfa (kg y^{-1})	Dimensionless	0.469	0.074	< 0.0001
Atmospheric deposition (kg y^{-1})	Dimensionless	0.387	0.045	< 0.0001
Manure from livestock production (kg y^{-1})	Dimensionless	0.110	0.037	< 0.0001
Crop fertilizer and fixation, other crops (kg y^{-1})	Dimensionless	0.180	0.113	0.1109
Developed land (km^2)	kg km^{-2} y^{-1}	2029.7	305.7	< 0.0001
**Land-to-water delivery**				
Northern Piedmont Ecoregion indicator (0,1)	Dimensionless	0.373	0.074	< 0.0001
Valley and Ridge Ecoregion indicator (0,1)	Dimensionless	0.535	0.078	< 0.0001
Average overland flow distance to stream channel (km)	km^{-1}	-0.225	0.030	< 0.0001
ln(ratio of nitrate to total inorganic nitrogen deposition)	Dimensionless	1.873	1.320	0.0785
Mean annual temperature (ln(°C))	Per ln(°C)	-0.712	0.124	< 0.0001
**Aquatic decay**				
**Streams**				
New England small streams ($Q_s \leq 2.83$ m^3 s^{-1})	m y^{-1}	50.92**	20.46	0.0133
New England large rivers ($Q_s > 2.83$ m^3 s^{-1})	m y^{-1}	4.381**	17.30	0.8016
Chesapeake Bay small streams ($Q_s \leq 2.83$ m^3 s^{-1})	m y^{-1}	50.17**	19.37	0.0100
Chesapeake Bay large rivers ($Q_s > 2.83$ m^3 s^{-1})	m y^{-1}	14.59**	12.49	0.2434
**Ponded waters**				
New England ponded waters	m y^{-1}	2.887**	2.408	0.2314
Chesapeake Bay ponded waters	m y^{-1}	3.568**	1.881	0.0560

**p*-values are one-sided for sources and aquatic decay (constrained to be non-negative) and two-sided for land-to-water delivery
**estimates of nitrogen uptake velocities (units of length per time)

Supplementary figures

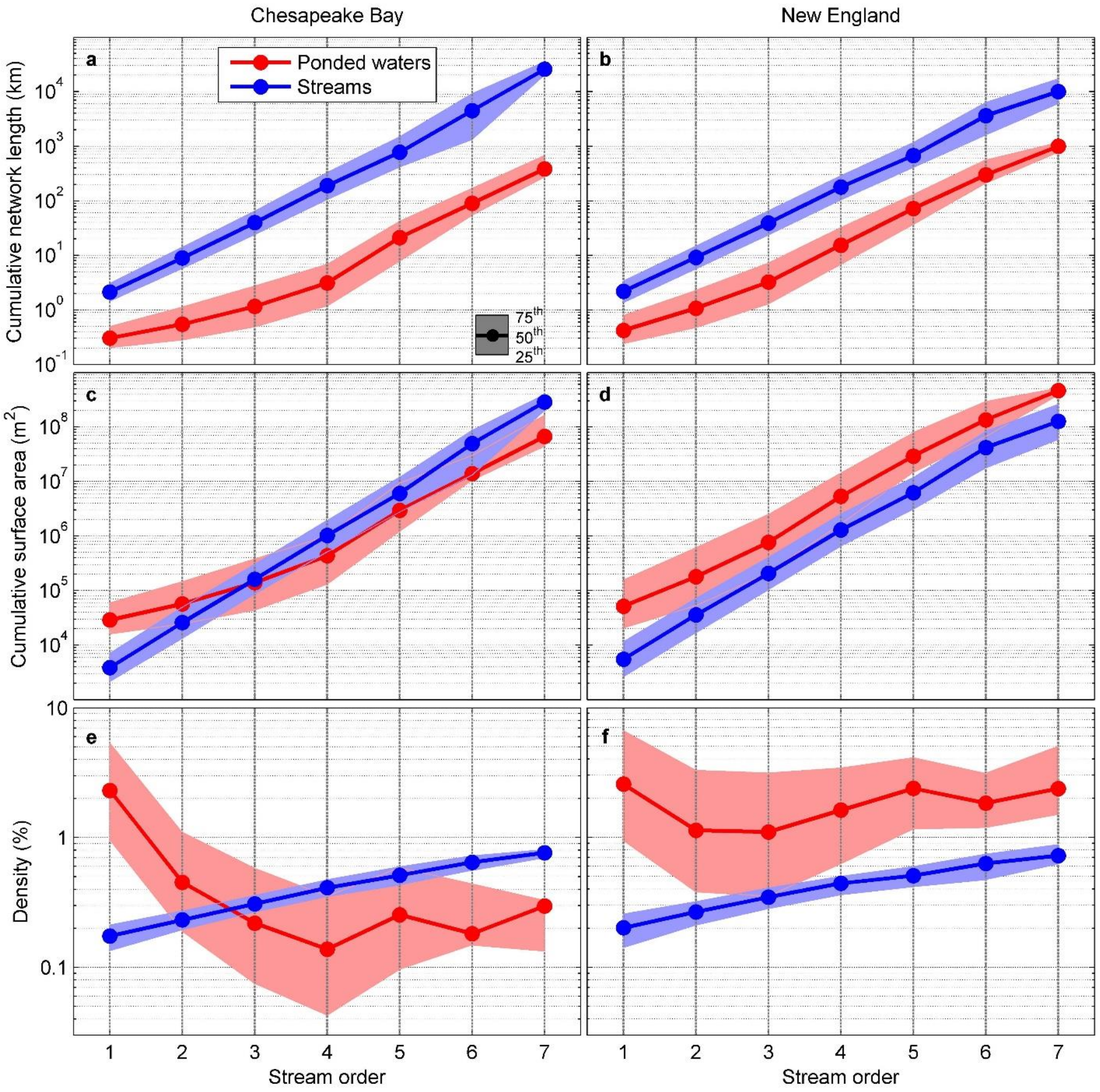

Supplementary Figure 1. Characteristics of streams and ponded waters of the existing river network. The left and right columns are of the Chesapeake Bay and New England sub-regions, respectively. Distributions (25th, 50th, and 75th percentiles) per stream order of the cumulative length of the river network covered by either ponded waters or streams (**a**, **b**), cumulative surface area of ponded waters and streams (**c**, **d**), and the density of ponded waters and streams as the cumulative surface area divided by cumulative drainage area (**e**, **f**).

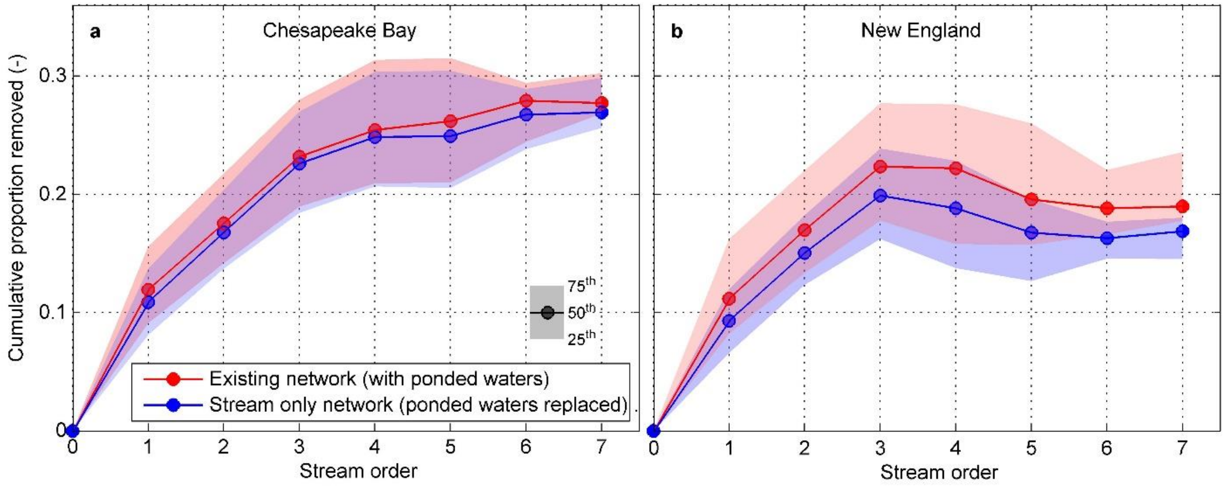

Supplementary Figure 2. Cumulative proportion of nitrogen removed in the existing river network (ponded waters and streams) and the river network with ponded waters replaced with streams. Distributions (25th, 50th, and 75th percentiles) are of the removal for the (**a**) Chesapeake Bay and (**b**) New England sub-regions. See Fig. 1a and 2 for the difference in cumulative proportion removed at every location throughout the river network.

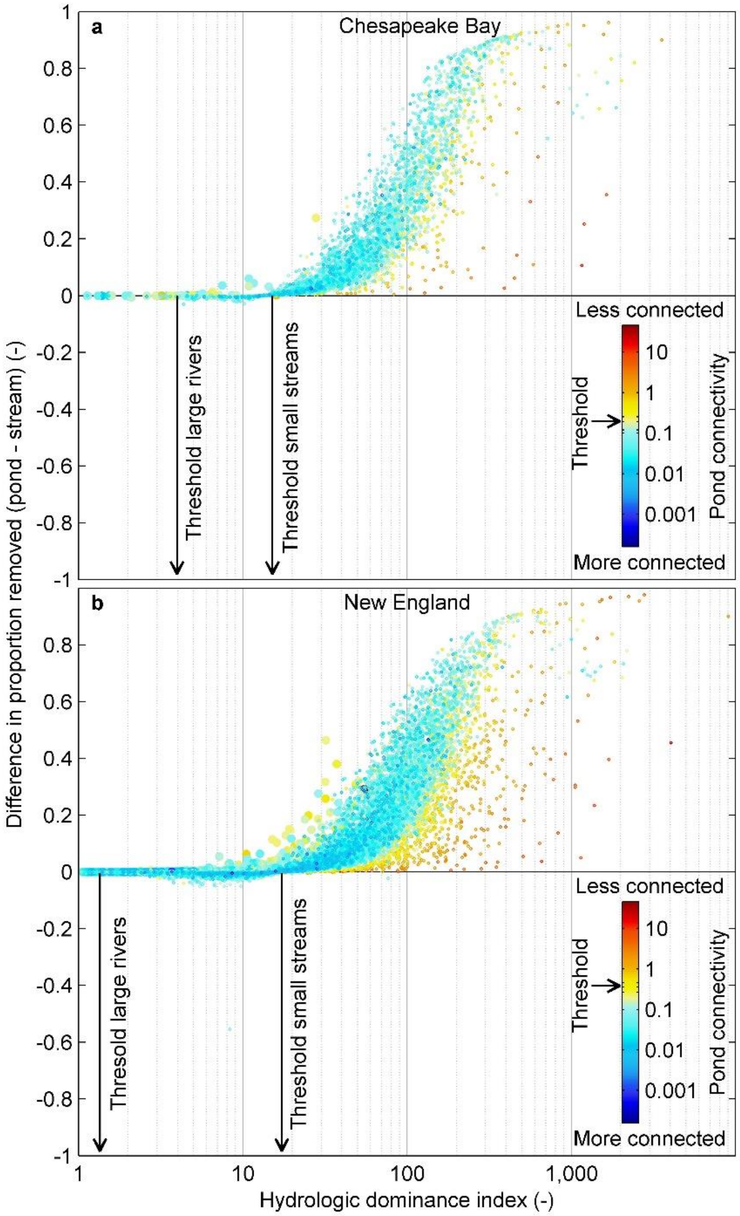

Supplementary Figure 3. Proportion of total nitrogen removed by an individual ponded water relative to its stream replacement (expressed as a difference) across varying degrees of pond connectivity. The results shown here are of the (**a**) Chesapeake Bay and (**b**) New England sub-regions for all ponded waters. The dot-plot is colored by varying degrees of pond connectivity (see equation (4)). The dominance of a ponded water to remove nitrogen starts to decrease at a threshold in pond connectivity of 0.19 (± 0.07) and 0.36 (± 0.09) for Chesapeake Bay and New England, respectively. The threshold in hydrologic dominance index where ponded waters becomes dominant occurs when it is equal to the ratio of stream to ponded water biological activity, which results from the case when the associated Damköhler numbers are equal (see equation (3)). There are two thresholds in the hydrologic dominance index because larger rivers have a lower biological activity than smaller streams (see Supplementary Table 5). The larger dots designate ponded waters on large rivers (mean annual discharge > 2.83 $m^3 s^{-1}$).

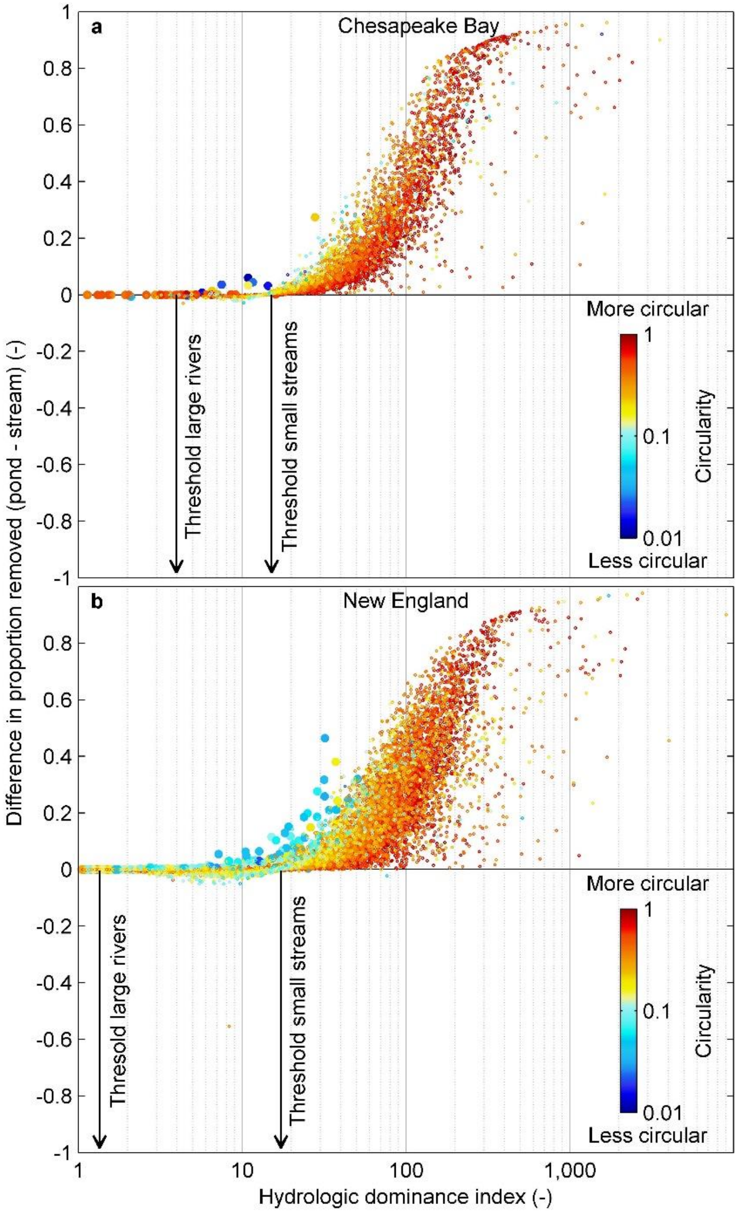

Supplementary Figure 4. Proportion of total nitrogen removed by an individual ponded water relative to its stream replacement (expressed as a difference) across varying degrees of pond shape. The results shown here are of the (**a**) Chesapeake Bay and (**b**) New England sub-regions for all ponded waters. The dot-plots are identical to Fig. S2, but are colored here by circularity (see equation (5)). A circularity approaching 1 indicates a more circular shape while approaching 0 indicates an increasingly elongated shape. The threshold in hydrologic dominance index where ponded waters becomes dominant occurs when it is equal to the ratio of stream to ponded water biological activity, which results from the case when the associated Damköhler numbers are equal (see equation (3)). There are two thresholds because larger rivers have a lower biological activity than smaller streams (see Supplementary Table 5). The larger dots designate ponded waters on large rivers (mean annual discharge > 2.83 $m^3 s^{-1}$).

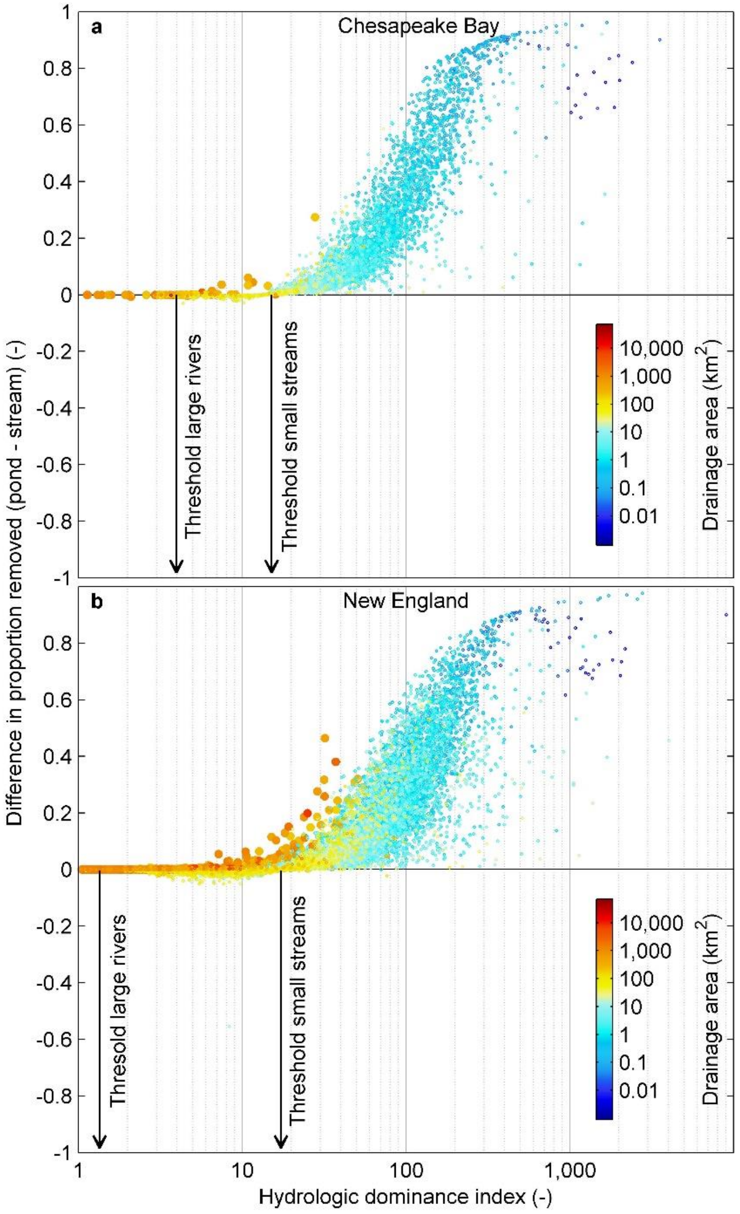

Supplementary Figure 5. Proportion of total nitrogen removed by an individual ponded water relative to its stream replacement (expressed as a difference) across varying degrees of drainage area. The results shown here are of the (**a**) Chesapeake Bay and (**b**) New England regions for all ponded waters. The dot-plots are identical to Fig. S2, but are colored here by cumulative upstream drainage area. The threshold in hydrologic dominance index where ponded waters becomes dominant occurs when it is equal to the ratio of stream to ponded water biological activity, which results from the case when the associated Damköhler numbers are equal (see equation (3)). There are two thresholds because larger rivers have a lower biological activity than smaller streams (see Supplementary Table 5). The larger dots designate ponded waters on large rivers (mean annual discharge > 2.83 $m^3 s^{-1}$).

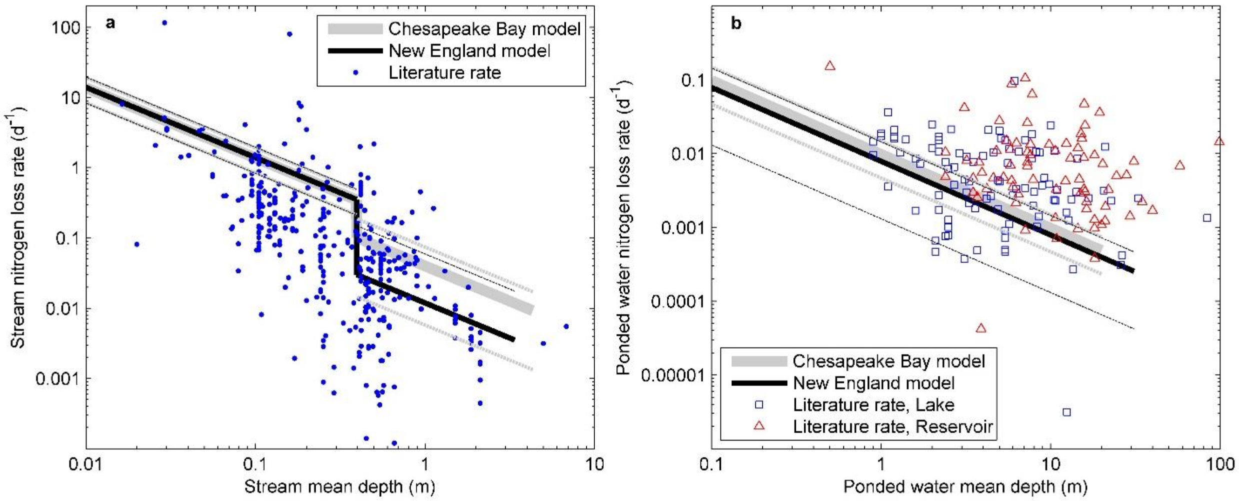

Supplementary Figure 6. Model and literature nitrogen removal rates. Model and literature estimates of the removal of total nitrogen for (**a**) streams and (**b**) ponded waters. Solid lines represent the mean uptake velocities and dashed lines represent the standard error about the mean (see Supplementary Table 5). Nitrogen loss rates are quantified as the uptake velocities divided by water depth. Literature values for streams are those compiled by ref. 1 and values for ponded waters are those compiled by ref. 2.

Supplementary References

1. Alexander, R. B. *et al.* Dynamic modeling of nitrogen losses in river networks unravels the coupled effects of hydrological and biogeochemical processes. *Biogeochemistry* **93,** 91–116 (2009).
2. Cheng, F. Y. & Basu, N. B. Biogeochemical hotspots: role of small water bodies in landscape nutrient processing. *Water Resour. Res.* **53,** 5038–5056 (2017).
